# Transcriptome Profiling of *Haloxylon persicum* (Bunge ex Boiss and Buhse) an Endangered Plant Species under PEG-Induced Drought Stress

**DOI:** 10.3390/genes11060640

**Published:** 2020-06-10

**Authors:** Fayas Thayale Purayil, Balaji Rajashekar, Shyam S. Kurup, Abdul Jaleel Cheruth, Sreeramanan Subramaniam, Nadia Hassan Tawfik, Khaled M.A. Amiri

**Affiliations:** 1 Department of Integrative Agriculture, College of Food and Agriculture, United Arab Emirates University, P.O. Box. Al-Ain 15551, UAE; fayas.t@uaeu.ac.ae (F.T.P.); abdul.jaleel@uaeu.ac.ae (A.J.C.); nadia.hassan@uaeu.ac.ae (N.H.T.); 2 Khalifa Center for Genetic Engineering and Biotechnology, United Arab Emirates University, P.O. Box. Al Ain 15551, UAE; 3 Institute of Computer Science, University of Tartu, 50409 Tartu, Estonia; balajior@gmail.com; 4 Celixa, Bangalore, Karnataka 560020, India; 5 School of Biological Sciences, Universiti Sains Malaysia (USM), Minden Heights, Georgetown, Penang 11800, Malaysia; sreeramanan@gmail.com; 6 Department of Biology, College of Science, United Arab Emirates University, P.O. Box. Al Ain 15551, UAE

**Keywords:** *Haloxylon persicum*, white saxaul, drought resistant, de novo transcriptome

## Abstract

*Haloxylon persicum* is an endangered western Asiatic desert plant species, which survives under extreme environmental conditions. In this study, we focused on transcriptome analysis of *H. persicum* to understand the molecular mechanisms associated with drought tolerance. Two different periods of polyethylene glycol (PEG)-induced drought stress (48 h and 72 h) were imposed on *H. persicum* under in vitro conditions, which resulted in 18 million reads, subsequently assembled by de novo method with more than 8000 transcripts in each treatment. The N50 values were 1437, 1467, and 1524 for the control sample, 48 h samples, and 72 h samples, respectively. The gene ontology (GO) and Kyoto encyclopedia of genes and genomes (KEGG) pathway analysis resulted in enrichment of mitogen-activated protein kinase (MAPK) and plant hormone signal transduction pathways under PEG-induced drought conditions. The differential gene expression analysis (DGEs) revealed significant changes in the expression pattern between the control and the treated samples. The KEGG analysis resulted in mapping transcripts with 138 different pathways reported in plants. The differential expression of drought-responsive transcription factors depicts the possible signaling cascades involved in drought tolerance. The present study provides greater insight into the fundamental transcriptome reprogramming of desert plants under drought.

## 1. Introduction

**Figure 1:**
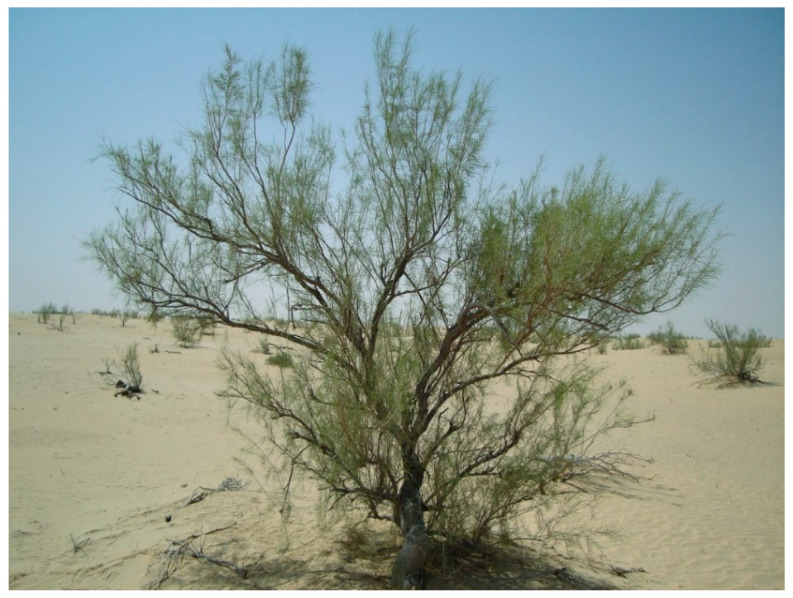
The *H. persicum* plant in its natural habitat, sand dunes.

*H. persicum* (Bunge ex Boiss and Buhse), the white saxaul belongs to the family *Amaranthaceae*, is an extremely drought-tolerant tree species with sand dunes as the habitat. It grows up to a height of 4 m. In the United Arab Emirates (UAE), *H. persicum* is generally found in gravel plains and sand dunes and has a great potential in landscaping. The tree is evergreen in the habitat with the leaves retrogressed as succulent branches of the tree. The plant has an extensive root system, making it useful for stabilizing sandy soils, especially in the desert ecosystem. The trees cover a desert land area of 1 million km^2^ across the Turanian deserts and appears in the hot deserts of the Middle East. This species is scattered throughout northwestern China and northern Russia [1]. Zohary, (1973) reported that in the Middle East the species is distributed in the Sinai Peninsula, Egypt, and central and northwestern Saudi Arabia on the Arabian Peninsula [2]. It has tremendous potential to be used as a landscape plant for urban landscaping and afforestation programs in the desert dunes of Central Asia, for combating desertification and land degradation in arid regions in respect to drought and thermal combating ability. Thus, the plant has been screened for landscape qualities, especially on its evergreen nature, compact growing form, and branching character, which produce a high canopy density. These trees are fast becoming endangered in respect to many factors, including climate change, unmanaged and over-grazing, faster urbanization, and other anthropogenic factors. As a xerophytic desert tree, *H. persicum* has tremendous drought and thermal tolerance, and thus serves a very crucial role in maintaining the ecosystem and the habitat in which it grows. These woodlands need to be conserved, since this is the only naturally occurring stand of the species in the United Arab Emirates and in eastern Arabia in general. The species has medicinal and economic importance as well. These woodlands are also microhabitats for many species of birds and small mammals, who require its fortification for survival (Figure 1).

In arid regions, drought is one of the major environmental restrictions that limits growth and productivity. During drought stress the plants undergo several changes, which eventually lead to the reduction in gas exchange and photosynthesis level and a decrease in cell division and cell expansion due to reduced enzyme activities and a lack of energy [3,4]. To adapt to these conditions, plants undergo various physiological, molecular, and cellular modifications to acclimatize with the changes imposed during the drought stress. These complex mechanisms of drought tolerance show variations among different plant species [5]. Functional proteins and regulatory proteins are two major groups of stress-inducible proteins, which play an important role in plant adaptation to stress conditions. Functional proteins are directly involved in plant defense under stress, which includes genes that are involved in the biosynthesis of osmo-protectants, membrane proteins, genes encoding chaperons, genes involved in growth and development, and genes encoding detoxification enzymes, etc. Regulatory proteins are involved in the regulation of downstream genes in a stress-response pathway. This includes different types of proteins kinases and transcription factors [6,7,8].

In plants, the abscisic acid-mediated signaling pathway plays a central role in plant stress responses [9,10]. Under a water deficit condition, the accumulation of abscisic acid (ABA) levels trigger the expression of ABA-responsive genes, leading to changes in physiological responses such as stomatal closure and other developmental process to alleviate the stress situation [11]. Under stress conditions, the regulation of stress-responsive genes is controlled by various transcription factors such as the bZIP family, the AP2/ERF superfamily, and the NAC family, etc. These transcription factors have significant roles in plant defense through ABA-dependent and ABA-independent stress-tolerance mechanisms [11,12]. Mitogen-activated protein kinase (MAPK) cascade is one of the major abiotic stress-response pathways involved in transducing external stimuli to the nucleus for appropriate adjustment of cellular responses under stress [13]. The role of MAPK-signaling cascade in abiotic stress, such as salt, osmotic stress, drought, heat, and cold, has been elucidated in different plant species [14]. Under stress condition, the phosphorylation of target genes are regulated by MAPK, which controls the activity of different structural proteins and various transcription factors which are involved in abiotic stress tolerance. Along with transcription factors (TFs), MAPK signal cascade also plays an important role in ABA-dependent and ABA-independent abiotic stress responses in plants [15]. Overall, various metabolic pathways and signaling molecules are involved in plants defense against abiotic stress. A balanced homeostasis of genes involved in various metabolic pathways are important for plant growth and development under stress [16].

Understanding the drought-tolerance mechanism is one of the critical steps towards the conservation and improvement of genetic resources for future. With the advent of next generation sequencing (NGS), efficient technologies are available for identifying the gene regulatory mechanism in plants. Deep ribonucleic acid (RNA) sequencing studies are achievable for all plant species by exploring transcriptome profiles of model and non-model organisms, under various conditions. These high-throughput sequencing techniques are widely used to create transcriptome data, which enables researchers to identify the differential expression profile of genes in plants under different treatment conditions [17,18,19]. In recent times, transcriptomic approaches have gained much attention for identifying the gene regulatory network associated with drought-stress tolerance in plants [20,21]. In this study, polyethylene glycol (PEG) 6000 (10%) was supplemented in culture medium to induce drought stress in *H. persicum* under in vitro conditions. Understanding the transcriptome profile under PEG-induced drought stress will enable us to identify the differential expression profile of genes under stress. Although some differences may occur between the responses to PEG-induced drought and the drought under field conditions, the present study will evidently provide greater insight into the regulatory networks associated with drought tolerance in these xerophytic plant species that can adapt to harsh climates of the arid region.

## 2. Materials and Methods

### 2.1. Plant Material and Drought Treatment

Two-year-old seedlings of *H. persicum* maintained in the greenhouse conditions, used for the study were provided by Environmental Agency, Abu Dhabi (UAE). Apical shoot bud explants collected from the seedlings were surface sterilized by washing initially with running tap water and then with 1% Tween 20 solution to remove any adhering contaminants. Under sterile conditions, the explants were treated with 20% sodium hypochlorite for 10 min and washed 4–5 times with sterile distilled water. The sterilized explants were inoculated onto a Murashige and Skoog (MS) medium and incubated at 25 ± 2 °C under 16 h photoperiod (3678 Lux) and 8 h dark conditions.

The one-month-old adventitious shoot buds of *H. persicum* cultures were treated with PEG 6000 10% (−0.60 MPa) in MS media for drought induction under in vitro conditions. The shoot buds were maintained at 16 h photoperiod and 8 h dark conditions at 25 ± 2 °C. Samples were collected at 48 h and 72 h of treatment under PEG media. The MS medium without PEG (−3.2 MPa) served as the control. Each treatment had three replicates and the samples were collected after the preferred incubation period. The water potential of the drought-treated and control samples were measured using a WP4C water potential meter (Meter, Pullman, WA, USA). The relative water content (RWC) was measured using the following equation [22]:

RWC=[(FW−DW)÷(TW−DW)]×100

where, *FW* is fresh weight, *DW* is dry weight and *TW* is turgid weight. *DW* is calculated after drying the samples for 24 h at 80 °C.

### 2.2. RNA Isolation and Transcriptome Library Preparation

The samples were collected from the PEG treatment at different periods of drought induction for RNA isolation and transcriptome profiling. The purification of RNA was carried out using a RNeasy mini kit (Qiagen, Germantown, MD, USA). The RNA concentration and purity were estimated using Nanodrop spectrophotometer (Thermo Fisher Scientific, Wilmington, DE, USA). The RNA integrity of the samples was checked using a Bioanalyzer (Agilent Technologies, Santa Clara, CA, USA). Briefly, 200 ng of RNA was used for mRNA isolation, fragmentation and priming. The first strand cDNA was synthesized in the presence of Actinomycin D, followed by second strand synthesis. The double stranded cDNA was purified using HighPrep PCR magnetic beads (Magbio Genomics Inc., Gaithersburg, MD, USA). Three replicates of each samples were pooled to form a single RNA sequencing library. The library was prepared with Illumina-compatible NEBNext^®^ UltraTM Directional RNA Library Prep Kit (New England BioLabs, Ipswich, MA, USA) based on the manufacturer’s instructions. Transcriptome sequencing was carried out using Illumina HiSEQ2500 at Genotypic Technology (Bangalore, India). The purified RNA was stored at −80 °C for real-time fluorescent quantitative PCR validation.

### 2.3. Illumina Raw Data Processing and Transcriptome Assembly

The raw illumina paired end (PE) read quality was assessed using FastQC tool [23]. Reads were trimmed by removing 3’ adapter, trimming ambiguous bases towards the read end and removal of PE reads, which had more than 30% of low quality (<q30) bases. Reads over 50 bp in length were retained after trimming process. Transcriptome de novo assembly was carried out using Trinity software, with the default k-mer value of 25 [24]. A Linux-based server with 256 GB of RAM and 20 cores was used for the transcriptome assembly. The redundant transcripts were clustered using CD-HIT program [25] to reduce redundancy and to obtain unigenes with sequence similarity (90%) and identity (95%) between the assembled transcripts. The clustered transcripts were used for further annotation with a minimum length of 300 bp.

### 2.4. Transcriptome Functional Annotation

The transcripts were annotated with the BlastX program (significant e-value of 6.738 × 10^−3^) [25] against NCBI Uniprot Viridiplantae sequences. The gene ontology information (Biological Process, Molecular Function and Cellular Component) associated with the transcripts was identified using the Blast2GO program [26]. Further Clusters of Orthologous Groups (COGs) classification was carried out using the NCBI-COG database [27]. Using the KEGG Automatic Annotation Server (KAAS) tool [28], transcripts were searched against the Kyoto encyclopedia of genes and genomes database (KEGG) [29] and KEGG orthology for identifying the metabolic pathways. During the pathway analysis, *Arabidopsis thaliana* (thale cress), *Tarenaya hassleriana* (spider flower), *Fragaria vesca* (woodland strawberry), *Theobroma cacao* (cacao), *Populus trichocarpa* (black cottonwood), *Glycine max* (soybean), *Citrus sinensis* (Valencia orange), *Cucumis sativus* (cucumber), *Vitis vinifera* (wine grape), *Solanum lycopersicum* (tomato) and *Oryza sativa* japonica (Japanese rice) were considered as reference organisms. Simple sequence repeats (SSRs) found in the clustered transcripts were identified using the MISA (http://pgrc.ipk-gatersleben.de/misa/) program, using the default settings.

### 2.5. Differential Gene Expression (DGE) Analysis

The fragments per kilobase per million fragments (FPKM) approach was used to calculate the gene expression. Transcripts from all the samples (control, T1 and T2) were pooled and clustered based on the similarity between transcripts using CD-HIT. The reads from different samples were aligned to the clustered transcriptome using the bowtie2 program [30], a pair of reads which supported each transcript were counted, and the significant gene expression difference was calculated using the DESeq tool [31] (FDR less than 0.01 were considered as a significant result). A heat map for differential expression was generated using the R program (https://www.r-project.org/).

### 2.6. qPCR Validation

The RNA-sequence differential gene expression was confirmed using qPCR. Ten genes that showed the significant differential expression during the DGE analysis were used for qPCR confirmation. The total RNA was converted into cDNA using an Affinity Script qPCR cDNA synthesis kit (Agilent Technologies, Santa Clara, CA, USA) as per the manufacturer’s protocol. In brief, 250 ng of RNA from each sample was taken for cDNA synthesis and the first strand cDNA was synthesized using universal oligo dT primers. The cDNA was diluted to 20 ng/µL and 1 µL of was used for each qPCR reaction. Specific qPCR primers were designed using the Primer3plus program (Supplementary Table S1). The expression levels of selected genes were analyzed using SYBR Green chemistry (Brilliant II SYBR Green qPCR master mix (Agilent Technologies, Santa Clara, CA, USA)) in Stratagene mx3005P instrument (Agilent Technologies, Santa Clara, CA, USA). The amplification cycling conditions were as follows: initial denaturation for 95 °C for 10 min, followed by 40 cycles of 95 °C for 30 s, 60 °C for 30 s. The dissociation curve analysis was performed after amplification for primer specificity; the conditions were as follows: 95 °C for 1 min, 55 °C for 30 s and 0.2 °C/s increment up to 95 °C (continuously collect fluorescence from 55–95 °C). Three replicates were included for each sample and the mean Ct value of the technical replicates was used to calculate the expression level. The relative quantification method (2^^-∆∆Ct^) was used to calculate the fold change using GAPDH as an internal control. The statistical significance between the treated samples were conducted by Student’s t-test using SPSS 16.0.

## 3. Results

### 3.1. Physiological Changes in H. persicum

**Figure 2:**
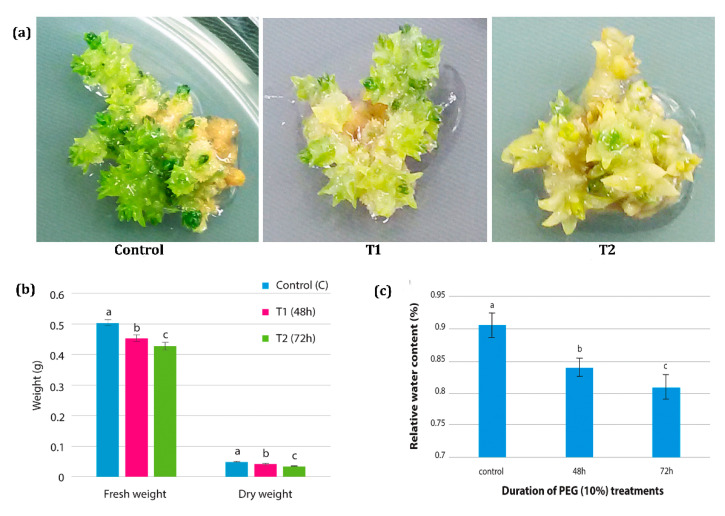
The effect of polyethylene glycol (PEG)-induced drought stress in *H. persicum*. (**a**) PEG-induced drought on adventitious shoot buds under in vitro conditions. Control: shoot buds at control media (MS without PEG), T1: cultures at 48 h of PEG treatment, T2: cultures at 72 h of PEG treatment. (**b**) The effect of PEG treatment on tissue fresh weight (g) and dry weight (g) in control sample (C), T1 sample (48 h PEG treatment) and T2 sample (72 h PEG treatment). (**c**) The effect of PEG treatment on Relative Water Content (RWC) in control samples, 48 h PEG treated samples and 72 h PEG treated samples. The error bar represents the standard deviation. Bar graph with different letters indicates significant difference between the mean values (*p* ≤ 0.05).

In the present study, in vitro drought stress studies in *H. persicum* was conducted by using 10% PEG 6000 (Figure 2a). Physiological parameters, such as fresh weight, dry weight, tissue water potential, and relative water content, were measured in all the treatment samples. The fresh weight and dry weight of the tissues showed significant changes under PEG treatment. There was a significant decline in fresh weight and dry weight observed when the shoot buds were subjected to PEG stress for 48 and 72 h. The fresh weight of the sample under control treatment was 0.505 g, which reduced significantly to 0.455 and 0.430 g in samples that are cultured on PEG medium for 48 h and 72 h, respectively. The dry weight of the tissues also showed gradual reduction upon increasing the PEG treatment period. The dry weight was 0.05 g under control condition, which reduced significantly to 0.0426 and 0.0353 g in tissues treated with PEG for 48 h and 72 h respectively (Figure 2b).

Relative water content and the tissue water potential (MPa) were monitored to confirm the influence of PEG treatment in imposing drought under in vitro condition. The relative water content of the samples showed significant changes under PEG treatment. While a maximum of 90% moisture content was observed in the control samples, under PEG treatments the relative water content was significantly reduced to 84% after 48 h. Due to the continued incubation of the cultures in the PEG treatment, the water content declined significantly to 81% after 72 h (Figure 2c). PEG 6000 treatment induced a significant reduction in tissue water potential. While the control samples without PEG showed −0.25 MPa water potential, after 48 h and 72 h of PEG treatment the tissue water potential significantly dropped to −0.55 MPa and −0.91 MPa, respectively.

### 3.2. Data Processing and Transcriptome Assembly

**Figure 3:**
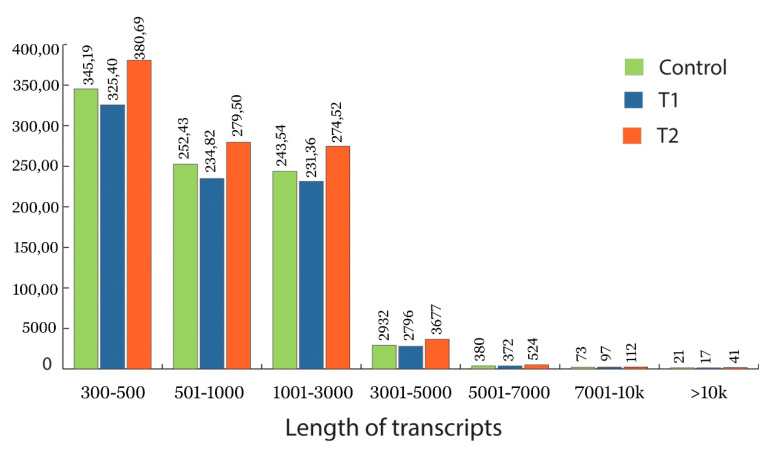
Length of the assembled transcript under different treatment conditions. Green represents the length distribution of the assembled transcriptome in the control samples of *H. persicum*, blue represents the length distribution of the assembled transcriptome in T1 (48 h PEG treatment), and red represents the length distribution of the assembled transcriptome in T2 (72 h PEG treatment).

The total RNA extracted from the control, T1, and T2 samples were converted into cDNA and sequenced using Illumina HiSeq2500 (151 × 2 bp chemistry). The transcriptome sequencing of the control, T1, and T2 libraries generated 19, 22.4, and 21.7 million reads (Table 1). The sequenced raw reads were deposited in the NCBI-SRA database (control SAMN08281247, T1 sample SAMN08281244, and T2 sample SAMN08281248). Read trimming resulted in 15.2, 18 and 21.7 million high quality reads (Q > 30) for the control, T1, and T2 libraries, respectively. The length distribution of assembled transcriptome is shown in Figure 3 for the control, T1, and T2 sample, respectively. The adenine (A), thymine (T), guanine (G) and cytosine (C) content of each sample are listed in the Table 1 and Supplementary Figure S1a–c. The initial assembly of the control samples resulted in 110,448 contigs; these contigs were further clustered into 87,522 transcripts with a N50 value of 1437 bp and GC percentage of 38.71, which represents 85.6 MB of the control sample transcriptome. De novo assembly of both the T1 and T2 libraries resulted in 105,017 and 123,307 contigs; the clustering of these contigs generated 82,440 and 97,825 transcripts. The assembly for the T1 sample generated 81.3 MB of transcriptome with the GC content of 38.92% and N50 value of 1467 bp. Similarly, the T2 sample generated 98.9 MB of transcriptome with a N50 value and GC content of 1524 bp and 38.54%, respectively (Table 1).

### 3.3. Transcriptome Annotation

**Figure 4:**
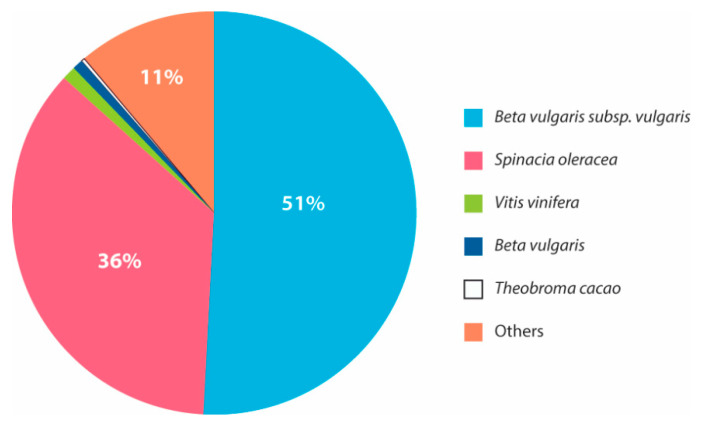
The percentage of similarity between the annotated *H. persicum* transcripts with other plant species.

A similarity search against NCBI and Uniprot plant protein databases resulted in 53.6% (46,992 transcripts), 55.1% (45,468 transcripts), and 51.6% (50,482 transcripts) of significant transcriptome annotations (e-value less than 6.738 × 10^−3^) in the control, T1, and T2 samples, respectively (Supplementary Tables S2–S4). A major portion of the annotated transcripts shared high similarity with the *Beta vulgaris* subsp., Vulgaris (~50%), *Spinacia oleracea* (~35%) and other plants (Figure 4). Further, the annotation resulted in the identification of important transcription factors that are involved in drought. The expression of the major transcription factors, such as NAC transcription factor (NAC), MYB family protein (MYB), dehydration-responsive element binding proteins (DREB), WRKY transcription factor family (WRKY), GATA transcription factors (GATA), and the basic leucine zipper domain (bZIP), were observed in all the samples sequenced.

### 3.4. Gene Ontology (GO) Classification, SSR Mining and Pathway Analysis

**Figure 5:**
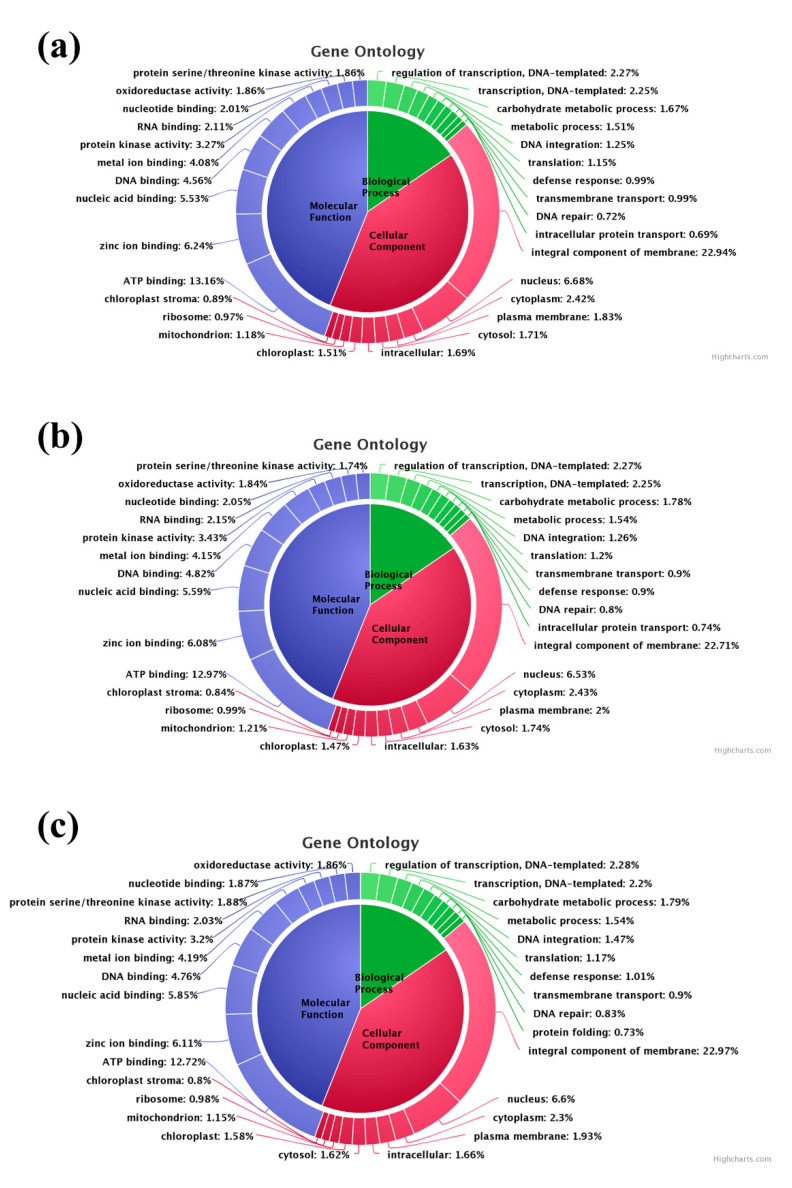
Gene ontology (GO) classification of the analyzed samples: (**a**) GO annotation in the control sample; (**b**) GO annotation in the T1 (48 h PEG treatment) sample; (**c**) GO annotation in the T2 (72 h PEG treatment) sample.

Gene ontology (GO) terms associated with each transcript were identified from Uniprot annotation based on the sequence homology. In the control sample, 26,253 Biological Process (BP), 24,148 Cellular Component (CC), and 44,004 Molecular Function (MF) related GO terms were identified (Figure 5a). In the T1 sample 25,490 BP, 23,494 CC, and 42,661 MF related GO terms were identified (Figure 5b). Similarly, in the T2 transcriptome 28,101 GO terms related to BP, 25,623 GO terms related to CC, and 46,873 MF related GO terms were identified (Figure 5c). Overall, the most expressed GO terms were GO:0016021 (integral component of membrane), GO:0005524 (ATP binding), GO:0005634 (nucleus), GO:0008270 (zinc ion binding), GO:0003676 (nucleic acid binding), GO:0003677 (DNA binding), GO:0046872 (metal ion binding), GO:0004672 (protein kinase activity), GO:0005737 (cytoplasm), GO:0005886 (plasma membrane), GO:0006355 (regulation of transcription, DNA-templated), and GO:0006351 (transcription, DNA-templated).

**Figure 6:**
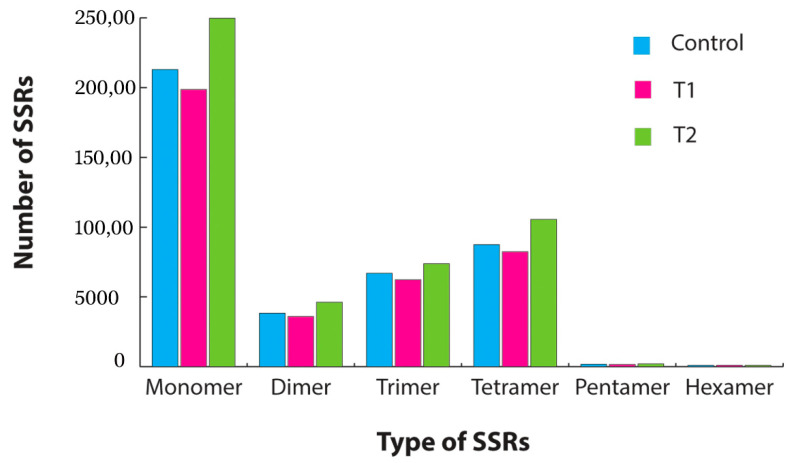
Simple sequence repeat (SSR) identification from the transcriptome data for each sample. Blue represents the identified SSR distribution in the control sample, pink represents the SSR distribution in the T1 (48 h PEG treatment) sample, and green represents the SSR distribution in the T2 (72 h PEG treatment) sample.

SSR mining was carried out using the MISA program. In total 19,548, 18,310, and 22,857 SSR repeats were identified in the control, T1, and T2 samples, respectively. From the overall samples, tetra repeats were found abundantly followed by tri and di repeats (Figure 6). The pathway analysis by KEGG-KAAS identified 138 plant-related metabolic pathways. In total, nearly 6200 transcripts functions were assigned to different pathways in all three samples. The major pathways identified in the transcriptome analysis were spliceosome(map03040), ribosome(map03010), carbon metabolism(map01200), protein processing in endoplasmic reticulum(map04141), plant–pathogen interaction(map04626), biosynthesis of amino acids(map01230), RNA transport(map03013), purine metabolism (map00230), plant-hormone signal transduction (map04075), mRNA surveillance pathway(map03015), endocytosis (map04144), pyrimidine metabolism (map00240), oxidative phosphorylation (map00190), RNA degradation (map03018), and ubiquitin-mediated proteolysis (map04120).

### 3.5. KEGG Analysis for Pathway Identification

**Figure 7:**
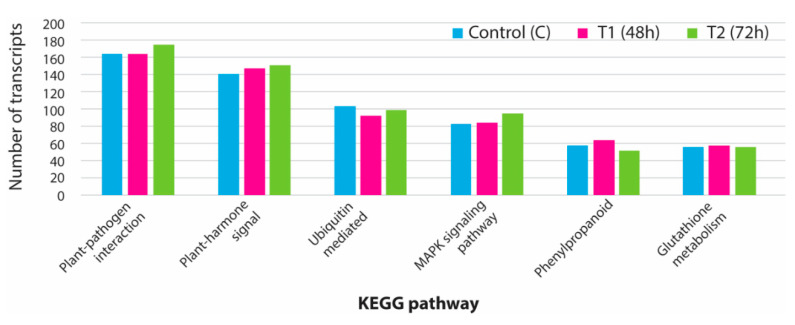
Total number of assembled transcripts associated with major pathways in plants mapped using Kyoto encyclopedia of genes and genomes database (KEGG). C: control; T1: 48 h PEG treatment; T2: 72 h PEG treatment.

The KEGG database was used to identify 138 important pathways involved in various developmental processes in plants. Some of the vital pathways, such as plant-hormone signal transduction pathways, plant pathogen interaction, MAPK signaling, phenylpropanoid biosynthesis, and ubiquitin-mediated proteolysis, were mapped with a significant number of expressed transcripts (Figure 7). From the assembled transcriptome data, we could find all the expressed genes mainly involved in the MAPK pathways. In total, 38 key genes involved in the MAPK pathway were identified (Supplementary Figure S2). The number of transcripts involved in the MAPK pathway varied from 83 to 94 transcripts in different sample groups. From the expressed transcriptome, 41 enzymes were found to be involved in plant-hormone signal transduction pathways. A significant number of transcripts were mapped against each plant-hormone-mediated signal cascade process (Supplementary Figure S3).

### 3.6. In Silico Differential Gene Expression (DGE) Analysis

**Figure 8:**
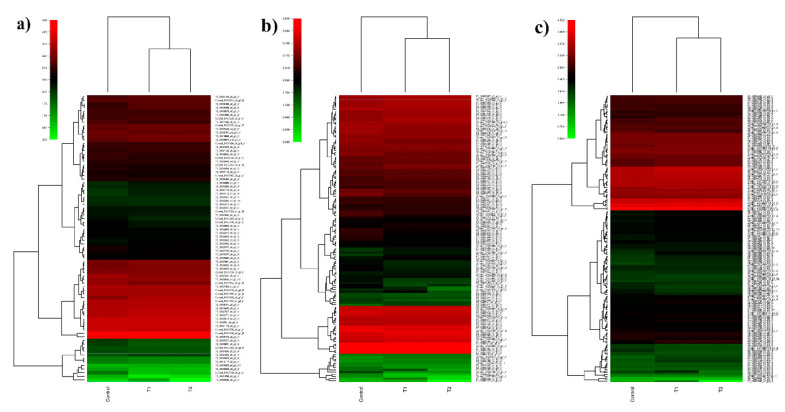
Differential gene expression pattern of selected KEGG pathway in *H. persicum*: (**a**) heat map showing the DGEs of MAPK-signaling genes in the control, T1 (48 h PEG treatment), and T2 (72 h PEG treatment) samples; (**b**) heat map showing the DGEs of plant-hormone signal transduction pathway genes in the control, T1, and T2 samples; (**c**) heat map showing the DGEs of plant pathogen interaction pathway genes in the control, T1, and T2 samples.

**Figure 9:**
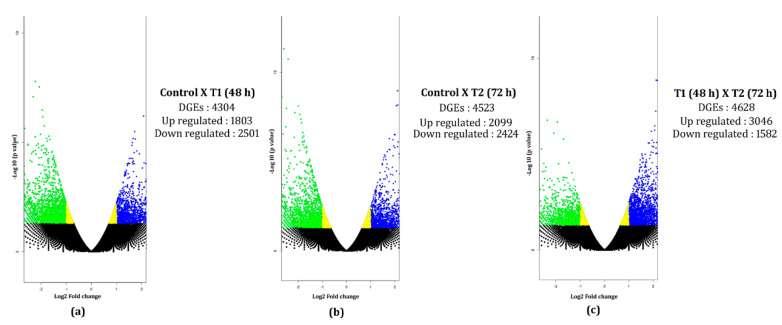
Volcano plots for the DEGs under different PEG treatments. Volcano plot DEGs generated for: (**a**) control (no drought) vs. T1 (48 h PEG treatment); (**b**) control (no drought) vs. T2 (72 h PEG treatment); (**c**) T1 (48 h PEG treatment) vs. T2 (72 h PEG treatment). The X and Y axis denote the log 2-fold change and -log10 (p-value), respectively. The blue dots represent upregulated genes, green represents downregulated, black represents the neutrally regulated.

**Figure 10:**
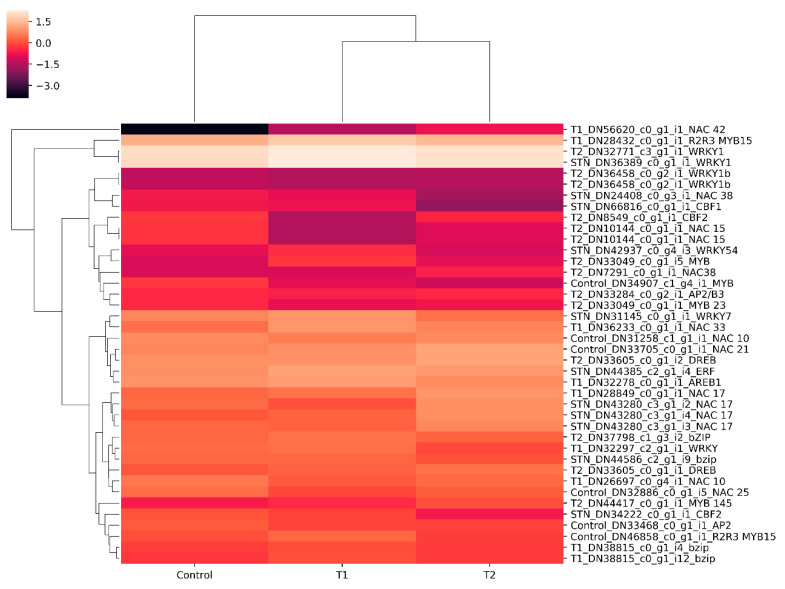
Differential gene expression patterns of transcription factors (TFs). Heat map showing differential expression of different TFs in the control, T1 (48 h PEG treatment), and T2 (72 h PEG treatment) samples.

Transcripts from the control, T1, and T2 samples were pooled and clustered using CD-HIT program, which resulted in 229,224 clustered transcripts. These clustered transcripts were considered as a reference for in silico DGE analysis. The read counts for all three samples were generated by aligning reads to the clustered transcripts using bowtie2, and normalized gene expression quantification was estimated using the DESeq program. Differentially expressed metabolically important enzymes were identified using KEGG database comparison (Figure 8). The DGE analysis between the control and T1 condition resulted in 1803 upregulated and 2501 downregulated transcripts (*p*-value < 0.01, Log2 fold change ±1). In the T2 samples, 3046 upregulated and 1582 downregulated transcripts were identified (*p*-value < 0.01, Log2 fold change ±1) (Figure 9). Approximately, 1153 transcripts showed significant differential expression in both T1 and T2 samples. Functional annotation for differentially expressed transcripts was performed by aligning against the Uniprot plant protein database. The differential expression of the transcription factors expressed under different treatments were represented as heat maps (Figure 10). Compared to other TFs, the transcripts of the NAC transcription factor showed higher expression under different treatment levels.

### 3.7. RT-PCR

**Figure 11:**
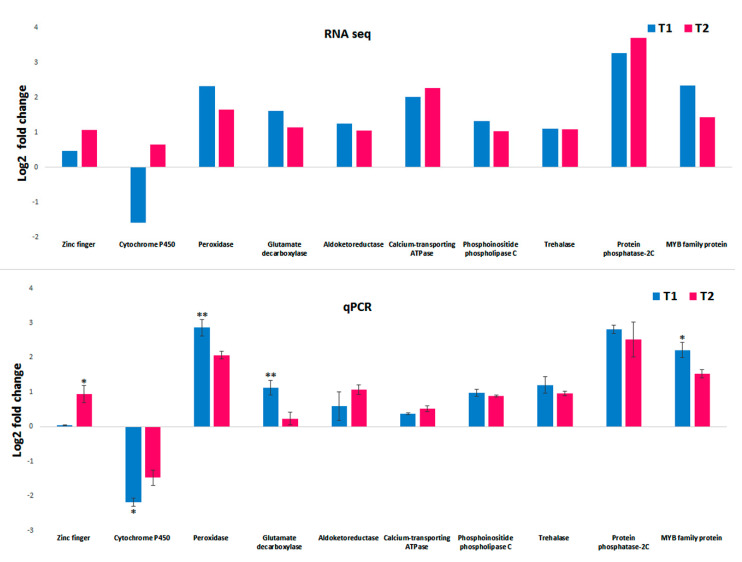
Validation of selected transcript from RNA seq using qPCR. The graph on the top shows the Log2FC of ten selected transcript DGE expressions in the T1 and T2 sample. The bottom graph represents the corresponding qPCR fold change in the T1 and T2 samples. The error bar represents the standard deviation. The difference between the means were calculated by Student’s t-test (*: *p* ≤ 0.05, **: *p* ≤ 0.01). T1: 48 h PEG treatment; T2: 72 h PEG treatment.

Quantitative real-time PCR analysis for the selected genes was performed to confirm the in silico DGE analysis data. From the transcript annotation, ten genes significantly expressed in both T1 (48 h PEG treatment) and T2 (72 h PEG treatment) compared to the control were selected for qPCR analysis. The analysis of ten genes, zinc finger (ZNF), cytochrome P450 (CYT), peroxidase (POX), glutamate decarboxylase (GAD), aldoketoreductase (AKR), calcium-transporting ATPase (CTAPSE), phosphoinositide phospholipase C (PI-PLCs), trehalase (TRE), protein phosphatase-2C (PP2C), and MYB family protein (MYB), showed positive correlations with the in silico DGE analysis data. The fold change trends in both the RNA sequence and the qPCR data are illustrated in Figure 11.

## 4. Discussion

In arid regions drought, salinity and thermal stress are the major hostile environmental factors that often limit crop production. Climate change and rapid urbanization are the potential factors that cause the erosion of biodiversity leading to the extinction of many species. *H. persicum*, an endangered nearly extinct species native to the arid region of the Middle East, has a great potential in urban landscaping considering its ability to combat thermal and drought stress. Therefore, identifying and conserving the drought-responsive genes from such endangered species has considerable significance for enriching the gene pool and future applications in drought studies. Drought stress and oxidative stress are often interrelated and may also exhibit identical and complexly adverse effects, leading to cellular damage. Exploring the expression profile of drought-responsive genes is essential for understanding the molecular mechanisms and metabolic pathways associated with drought-stress tolerance in crops [6,32].

The focus of the present study was to identify the upregulated and downregulated genes involved in the growth and maintenance of adventitious shoot buds developed in vitro under PEG-induced drought. PEG 6000 (100 g L^−1^) was used in the media to create a rapid drought stress through water deprivation [33]. A significant decline in fresh weight, dry weight, RWC, and tissue water potential (Mpa) along with changes in the phenology of the adventitious shoot buds confirmed the PEG-induced drought stress. Transcriptional reprogramming under stress is one of the major mechanisms that plants undergo during stress tolerance. In the present study, transcriptome profiles of the two drought-induced (PEG) samples T1 (48 h) and T2 (72 h) were compared with the control samples without any drought treatment. This resulted in 82,440 assembled transcript (T1) with an average length of 978 bp (control). The N50 lengths were 1437, 1467, and 1524 bp for the control, T1, and T2, respectively. These findings are comparable with the results of RNA-sequence in *Haloxylon ammodendron* (N50: 1345 bp) under drought stress [34].

During drought stress, significant changes in gene expression occur in plants in order to survive under drought by regulating cellular process [35]. Elucidating the drought-stress tolerance in plants by differential gene-expression methods provides valuable information for identifying probable stress tolerance mechanism. The DGE analysis displayed the differential expression of approximately 1153 transcripts in both T1 and T2 samples, which was relatively higher in number compared to *H. ammodendron* transcriptome [34]. In the present study, the number of DEGs showed a significant change when the PEG stress was increased from 48 h to 72 h. Similar findings were reported in cassava, where a significant changes observed in DEGs when the PEG stress period was increased from 3 h to 24 h [36]. In addition, our results indicate that the differential expression of downregulated genes showed gradual reduction in number upon increase in the PEG stress period, whereas the upregulated genes showed a steady increase in number under elevated stress period. Overall, the percentage of downregulated genes was higher in both treatment conditions (Figure 9). These results were in alignment with the findings of Liu et al. (2014), and Ma et al. (2015), who reported that the abundance of downregulated DGEs was significantly higher compared to upregulated genes under PEG stress [16,17].

The transcription factors are regulatory proteins which play a major role in drought tolerance by synchronizing the signaling network and gene expression under stress [37]. NAC transcription factors are ubiquitous in plants, and their expression shows significant changes under abiotic stresses [38]. In our dataset, the expression of the NAC transcription factor (DN33705_c0_g1_i1) gene was upregulated under PEG stress, compared to the control (Figure 10). The expression level elevated significantly when the PEG treatment period shifted from 48 h to 72 h. The involvement of the NAC TFs in drought tolerance has been demonstrated in various crops like rice [39], and soybean [40]. Dehydration responsive element binding factor (DREB) is an AP2/ERF-type transcription factor present in plants [41]. The overexpression of the OsDREB2B gene in Arabidopsis resulted in improved tolerance to drought stress [42]. In the present study, DREB (DN33605_c0_g1_i2) transcripts displayed significant upregulation under PEG-induced stress; this denotes the probable role of DREB TFs in the downstream process of the drought-tolerance mechanism in *H. persicum*. Other drought-responsive TFs, such as MYB, WRKY, bZIP, bHLH, and C2H2, showed differential expression under PEG-induced drought stress, as reported in other plant species [43].

The GO enrichment analysis was performed to predict the role of transcripts in the biological, cellular, and molecular process of the cells. In all the three sample groups, more than 50% of the DEGs were involved in various GO terms. Drought-induced genes are functionally classified as two major groups, namely functional proteins and regulatory proteins. Functional proteins are involved in stress tolerance and the regulatory proteins have a major role in stress response through alteration gene expression and signal transduction [7]. The KEGG pathway analysis resulted in mapping the DEGs with 138 different pathways that were involved in various cellular processes. The MAPK cascades are a multigene family of regulatory networks deployed for the transduction of intra- and extra-cellular signals to the nucleus for suitable adjustments of the cell to the stimuli [13]. In this study, 38 potential genes were mapped with the MAPK pathway, which includes the expression of all the three components of MAPK cascade (MAPKKK, MAPKK and MAPK). MAP kinases that are involved in ethylene, jasmonic acid, and abscisic acid-mediated stress responses are significantly mapped in the present study (Supplementary Figure S2).

**Figure 12:**
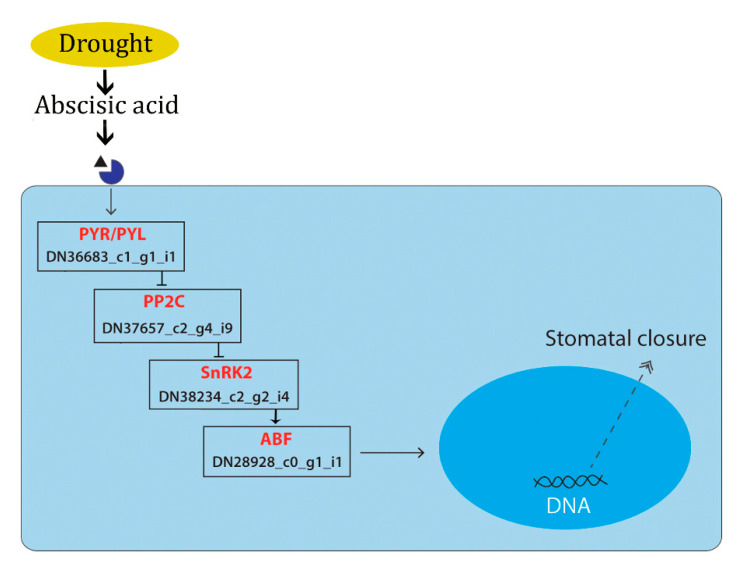
Schematic representation showing the transcript expressed under PEG-induced drought stress mapped in the ABA-mediated KEGG pathway. The genes expressed in the present study are given in red font and the transcript ID is given in black font.

Phytohormones play a significant role in plant stress regulation. During drought induction, the environmental stress factors induce the secretion of plant hormones which mediates the immediate cell responses by triggering plant hormone signal transduction pathways. Studies show that abscisic acid exhibit an important role in the drought tolerance of plants through stomatal closure and the activation of the stress-responsive genes [7]. In the present study, under PEG-induced stress, the ABA-mediated drought response facilitated by PP2C gene (Transcript Id: T2_DN28928_c0_g1_i1) was mapped, which in turn triggered the ABF (ABA-responsive element binding factor) (DN22654_c0_g1_i1) gene, which plays a major role in stomatal closure (Figure 12). Similar responses were reported in *Phormium tenax* [44]. Reports suggests that, the overexpression of abscisic acid stress ripening (ASR) gene family play a significant role in drought and salinity stress [45]. We found upregulation of ASR gene (DN23961_c0_g2_i3) was observed under PEG induced drought stress, which indicates the possible role of ASR genes in ABA-mediated stress tolerance. Increased stress tolerance in response to the overexpression of ASR genes was reported in transgenic plants under various stress conditions [46,47,48,49].

In the present study, the ethylene-mediated plant-hormone signal transduction pathway was also explored. Ethylene is an important plant-growth regulator which regulates the plant growth under drought condition by undertaking various developmental changes in the plant [50]. Mapping the DEG for ethylene-responsive transcription factor (ERF) 1 in the KEGG pathway denotes the possible activation of ethylene-induced defense mechanism under PEG-induced stress conditions. The role of ERFs in the developmental process such as cuticle biosynthesis was reported earlier in Arabidopsis [51]. The crosslink between the jasmonic acid and ethylene-mediated pathway observed in the MAPK-signaling cascade, shows the possible role of jasmonic acid signaling intermediates in ethylene-induced defense response (Supplementary Figure S2). These findings are in accordance with the observation of Lorenzo et al. (2002), who reported that the ERF1 expression could be regulated by ethylene and jasmonic acid or the synergistic effect of both hormones [52].

The accumulation of reactive oxygen species (ROS) is one of the major modifications to occur under drought or salt stress, causing damages to the cell membranes [53]. Antioxidant enzymes play a crucial role in drought-defense mechanisms by scavenging ROS generated under drought stress [54]. Peroxidase is an important antioxidant enzyme involved in the detoxification of H_2_O_2_ molecules generated under stress. In our study, 30 DEGs matching the expression of peroxidase (EC 1.11.1.7) enzyme were identified. The expression of the enzyme was greater in the T2 sample (72 h) compared to the T1 (48 h) treatment. This shows the increased accumulation of peroxidase enzyme with a concomitant increase in PEG-induced drought stress. A similar pattern of expression changes in genes associated with peroxidases have been reported in many different plant species under drought stress [55]. Several other antioxidant enzymes, such as superoxide dismutase (SOD), catalase (CAT) and glutathione s-transferase (GST), displayed an upregulated DEG profile, which confirms the biochemical rearrangements in *H. persicum* under the PEG-induced drought stress condition.

## 5. Conclusions

In this study, we investigated the transcriptome profile of *H. persicum*, an endangered desert plant species. This is the first report on this plant, and we reported ~50,000 annotated transcripts and their possible metabolic pathways under PEG-induced drought stress. In this analysis, we identified the expression pattern of drought-responsive genes using differential gene expression analysis, which resulted in a minimum of 4304 DEGs under 48 h PEG treatment and a maximum of 4523 DEGs under 72 h PEG treatment. Elucidating the pathway results revealed the possible defense mechanism under PEG-induced drought in *H. persicum*. The present study will provide a better understanding of drought-response mechanism of the desert plant under stress conditions. Some differences may occur between the responses to PEG-induced drought and the drought under field conditions; nevertheless this provides valuable evidence to confirm the transcriptional reprogramming of genes under stress.

## Figures and Tables

**Table 1:** *H. persicum* transcriptome assembly statistics for the control sample, T1 (48 h PEG treatment) sample and T2 (72 h PEG treatment) sample.

	Control	T1	T2
Total raw reads (PE reads)	19,007,972	22,409,657	21,771,386
Total trimmed reads (PE reads) in Million	15.24	18.04	17.12
Read length (bp)	151	151	151
Total transcripts	87522	82440	97825
Total bases	85,611,629	81,373,840	98,916,209
(G + C) %	38.71	38.92	38.54
N%	0	0	0
Minimum sequence length	300	300	300
Maximum sequence length	16,107	16,067	16,736
Average sequence length	978.17	987.07	1011.15
N50 length	1437	1467	1524
L50 number	17,792	16,679	19,400

## Data Availability

The datasets generated are available in NCBI-SRA database with samples IDs for control SAMN08281247, T1 sample SAMN08281244, and T2 sample SAMN08281248.

## Supplementary Materials

The following are available online at https://www.mdpi.com/2073-4425/11/6/640/s1, Supplementary Table S1: List of primers used for qPCR validation; Table S2: Transcriptome annotation file for control sample; Table S3: Transcriptome annotation file for T1 sample (48h of PEG stress); Table S4: Transcriptome annotation file for T2 sample (72h of PEG stress); Figure S1: Percentage of ATGC content in H. persicum. (a) ATGC content in control sample, (b) ATGC content in T1 sample (c) ATGC content in T2 sample; Figure S2: KEGG pathway genes mapped for MAPK signaling in H. persicum under PEG induced stress (Red box denotes genes expressed in the present study); Figure S3: KEGG pathway genes mapped for plant hormone signal transduction in H. persicum under PEG induced stress. (Red box denotes genes expressed in the present study).
