# Supplementary material for: Transcriptome Profiling of Haloxylon persicum (Bunge ex Boiss and Buhse) an Endangered Plant Species under PEG-Induced Drought Stress
Source: Genes (Basel). 2020 Jun 10;11(6):640. doi: 10.3390/genes11060640 (PMC7349776; doi:10.3390/genes11060640)
Supplement: Supplementary file 1 [file genes-11-00640-s001.zip › genes-807502-SI/Supplementary table S1.docx]

| Sl. No | Primer ID | Primer Seq 5'---- to 3' | Primer size (bp) |
| --- | --- | --- | --- |
| 1 | ZNF | CTTTGCTGAGGATGGAGCAC  AACTGCAGACACATCAACCG | 20 |
| 2 | CYT | ACAGAGCTCCTACACAACCC  AACAGTTGGTGGGTGCATTC | 20 |
| 3 | POX | AGAAGAGGCTTGAACACCGA  GTTGGGTCTGATGTCTGGGA | 20 |
| 4 | GLD | GCGATTCACCATTGTGTCCA  TAACCACAACTCGGAGCACT | 20 |
| 5 | AKR | AGGTCTTGGATGCATGGGAA  GTGTCAAGGAAGGTGATGCC | 20 |
| 6 | CTPASE | GGTGGTATCAGTTGGCTCCT  TGAGGGCTAGTCAGCATGAG | 20 |
| 7 | PI-PLCs | AAATCAACCCTTGGCAACTG  CAGCGGAACCTTTTCTCATC | 20 |
| 8 | TRE | AGAATCAGGCAAAGGAAGCA  GCCAAATGGGTTTTTACCAA | 20 |
| 9 | PP2C | ACGCGAACCTTCCCTTTACT  ACTTGCCGGAGAAACATCAC | 20 |
| 10 | MYB | CTGATGAGGAGAAGGGCTTG  AGGAGGTGCTGGAGATTGTG | 20 |
| 11 | GAPDH | GGCAAGGTGCTTCCATCATT  TTTGAGGTTGCCCTCAGACT | 20 |

**Primer list used for qPCR validation**
