# Supplementary figures and images for: Transcriptome Profiling of Haloxylon persicum (Bunge ex Boiss and Buhse) an Endangered Plant Species under PEG-Induced Drought Stress
Source: Genes (Basel). 2020 Jun 10;11(6):640. doi: 10.3390/genes11060640 (PMC7349776; doi:10.3390/genes11060640)

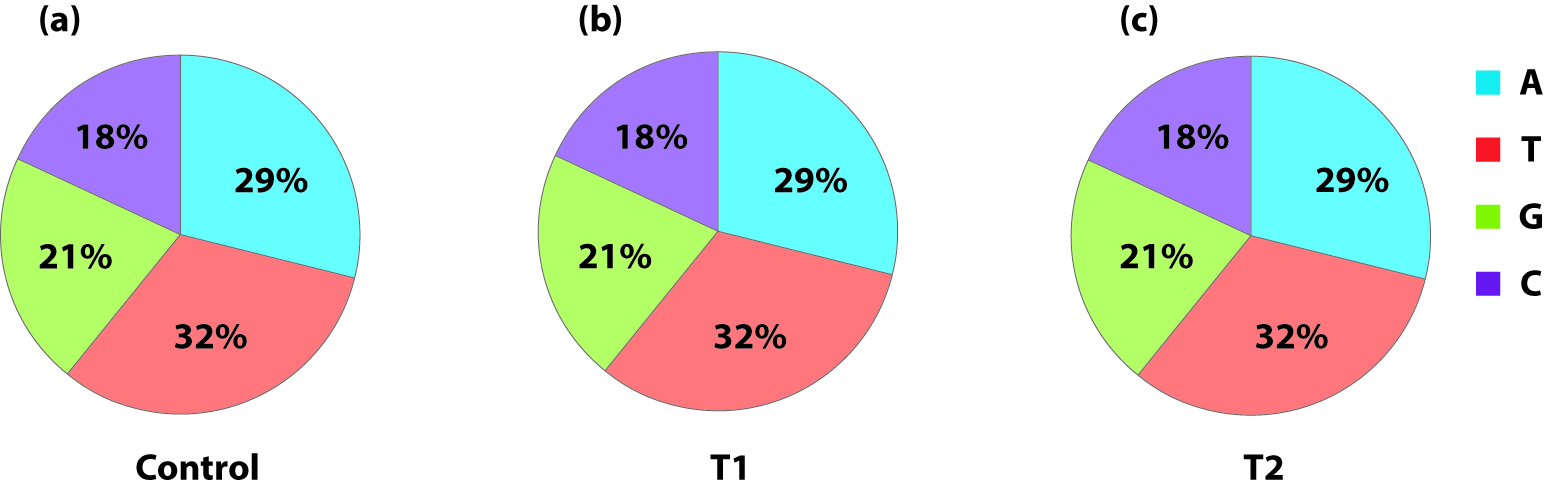

Supplement: Supplementary file 1 [file genes-11-00640-s001.zip › genes-807502-SI/Suppl_Figure 1.tif]

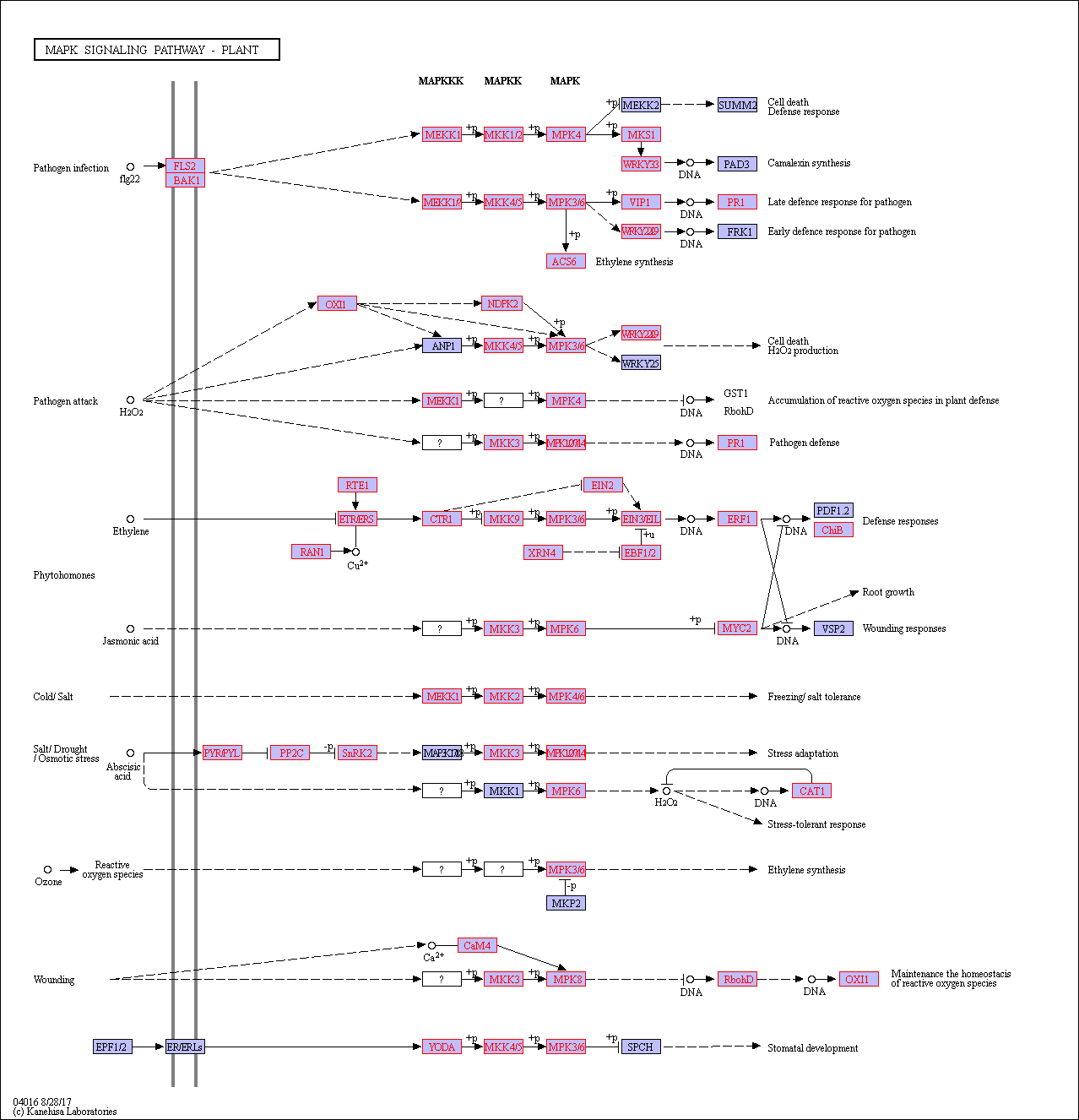

Supplement: Supplementary file 1 [file genes-11-00640-s001.zip › genes-807502-SI/Suppl_Figure 2.tif]

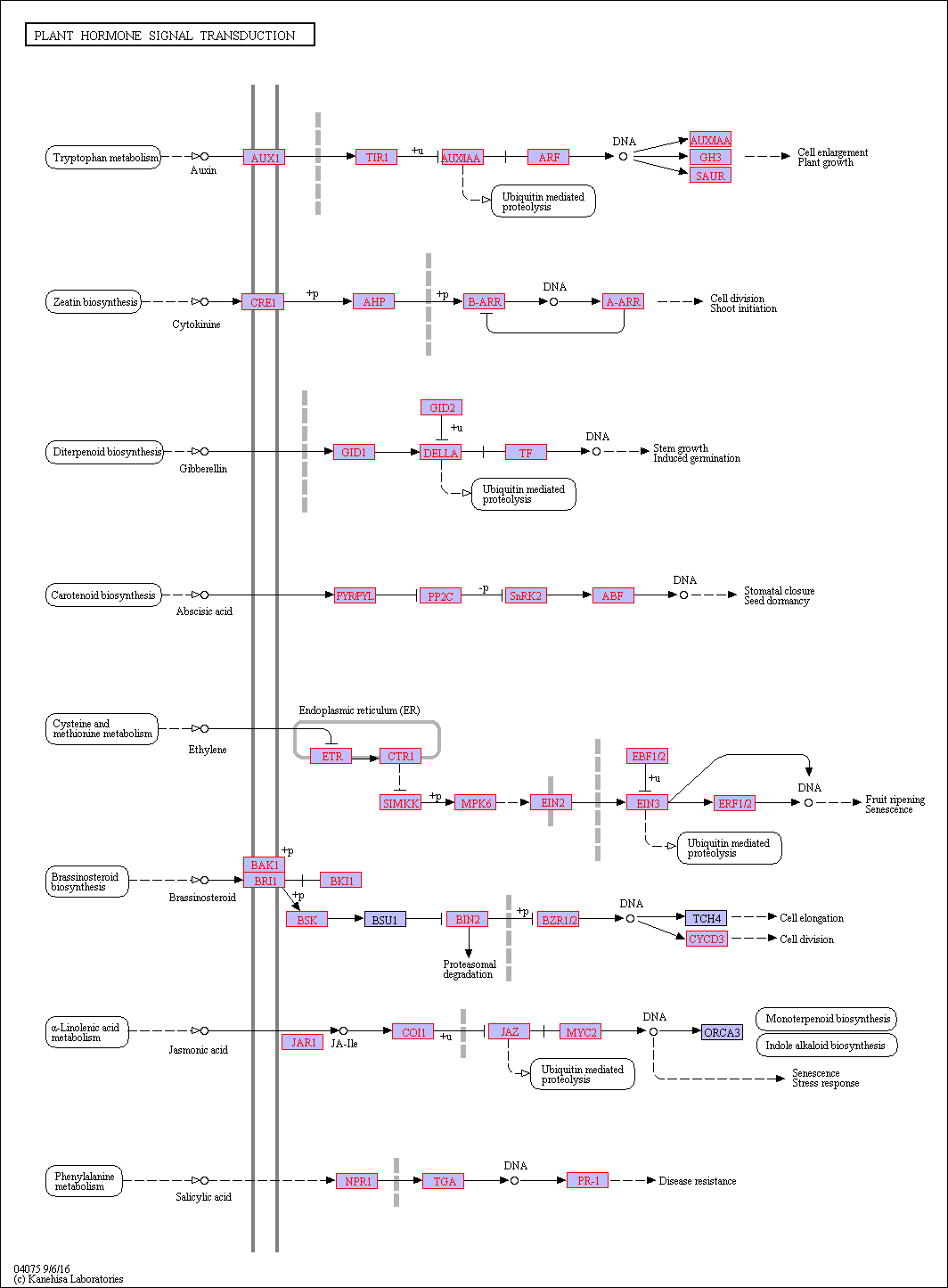

Supplement: Supplementary file 1 [file genes-11-00640-s001.zip › genes-807502-SI/Suppl_Figure 3.tif]
